# Supplementary material for: Adenosine triphosphate-activated prodrug system for on-demand bacterial inactivation and wound disinfection
Source: Nat Commun. 2022 Aug 11;13:4712. doi: 10.1038/s41467-022-32453-3 (PMC9372092; doi:10.1038/s41467-022-32453-3)
Supplement: Supplementary file 1 — Supplementary Information [file 41467_2022_32453_MOESM1_ESM.pdf]

Supplementary Information

Adenosine triphosphate-activated prodrug system for on-demand bacteria inactivation and wound disinfection

Yuhao Weng[1], Huihong Chen[1], Xiaoqian Chen[1], Huilin Yang[2], Chia-Hung Chen[3], Hongliang Tan[1*]

1. National Engineering Research Center for Carbohydrate Synthesis/Key Lab of Fluorine and Silicon for Energy Materials and Chemistry of Ministry of Education, College of Chemistry and Chemical Engineering, Jiangxi Normal University, Nanchang 330022, PR China
2. College of Life Science, Jiangxi Normal University, Nanchang 330022, PR China
3. Department of Biomedical Engineering, City University of Hong Kong, Tat Chee Avenue, Kowloon, Hong Kong SAR, PR China

* Corresponding author: hltan@jxnu.edu.cn

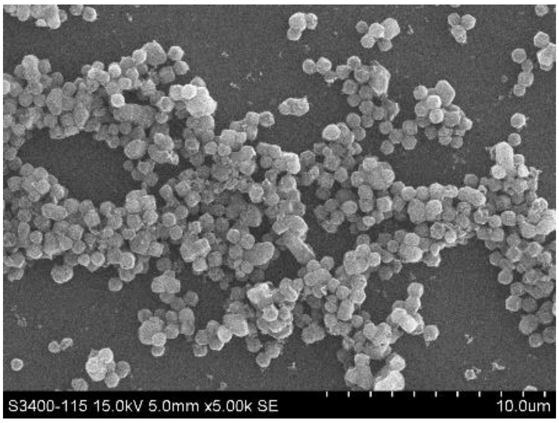

Supplementary Figure 1. SEM image of ZIF-8. The SEM image is representative of three independent experiments with similar results.

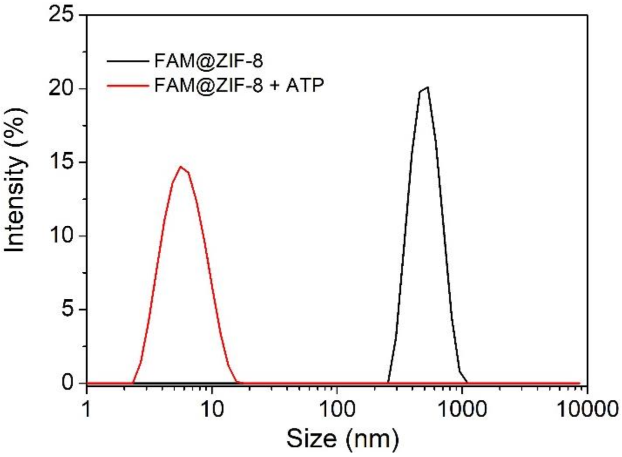

Supplementary Figure 2. Hydrodynamic diameters of FAM@ZIF-8 before and after treating with ATP.

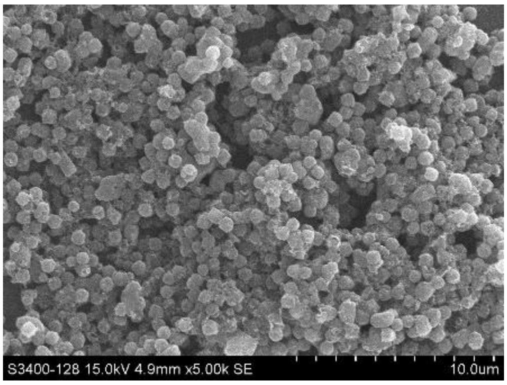

Supplementary Figure 3. SEM image of IAA@ZIF-8. The SEM image is representative of three independent experiments with similar results.

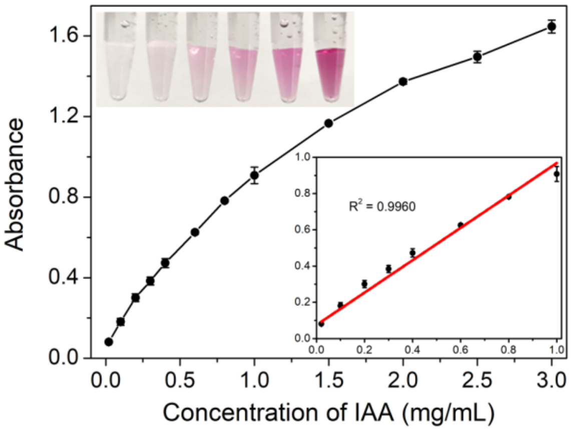

Supplementary Figure 4. Absorption intensity of Salkowski reagent in the presence of IAA with different concentrations. Inset is the linear calibration plots of absorption intensity at 529 nm against IAA concentration. Data are presented as mean values ± SD (n = 3 independent samples).

Salkowski reagent is a mixture of 0.5 M ferric chloride ($FeCl_3$) and 35% perchloric acid ($HClO_4$), which upon reaction with IAA yields pink color, due to IAA complex formation with and reduction of Fe^{3+}. Typically, the colorimetric Salkowski assay was performed by adding IAA with different concentrations (from 0 to 3 mg/mL) to 150 μL of Salkowski reagent for reacting 30 min at room temperature. The final volume of this colorimetric assay was kept at 300 μL by adding PBS buffer (10 mM, pH7.4).

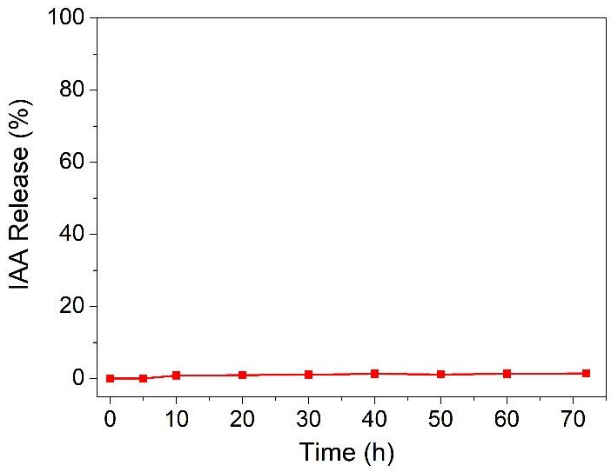

Supplementary Figure 5. Release profile of IAA from IAA@ZIF-8 in PBS buffer. Data are presented as mean values ± SD (n = 3 independent samples).

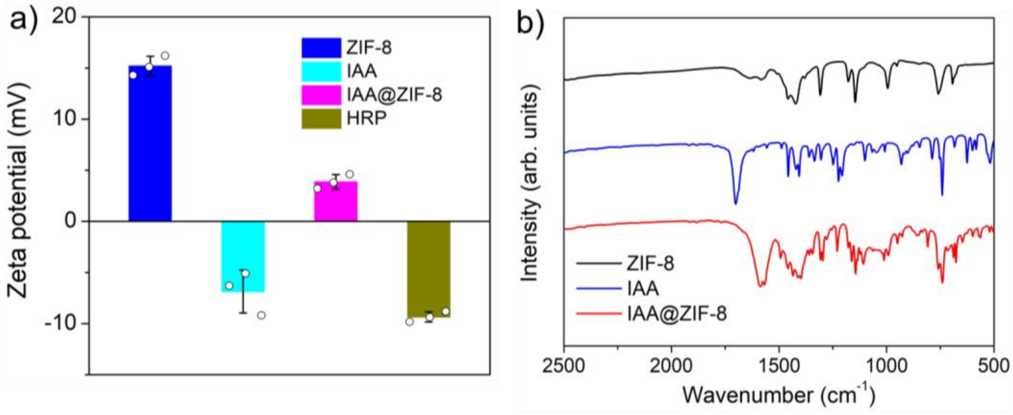

Supplementary Figure 6. (a) Zeta potential of ZIF-8, IAA, IAA@ZIF-8, and HRP. Data are presented as mean values ± SD (n = 3 independent samples). (b) FTIR spectra of IAA, ZIF-8 and IAA@ZIF-8.

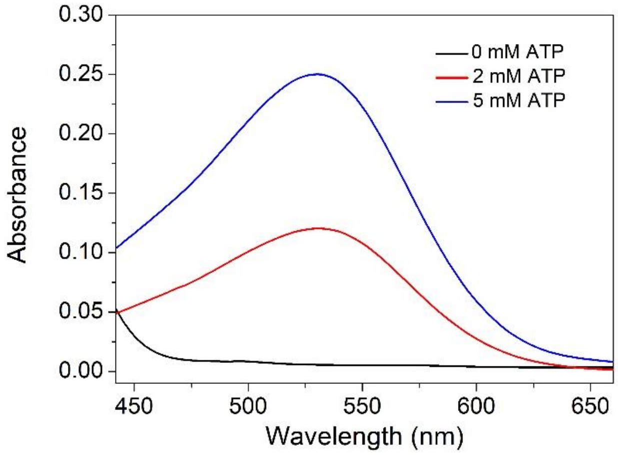

Supplementary Figure 7. Absorption spectra obtained from the reaction of Salkowski reagent with the supernatant of IAA@ZIF-8 after treating with different concentrations of ATP.

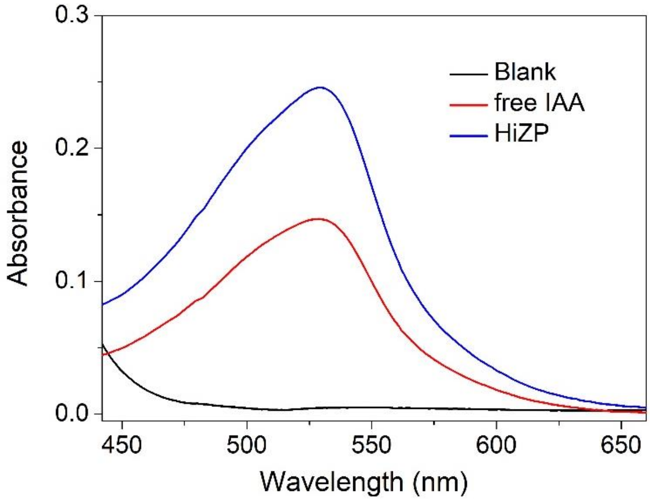

Supplementary Figure 8. Absorption spectra obtained from Salkowski reagent in the presence of free IAA and HiZP.

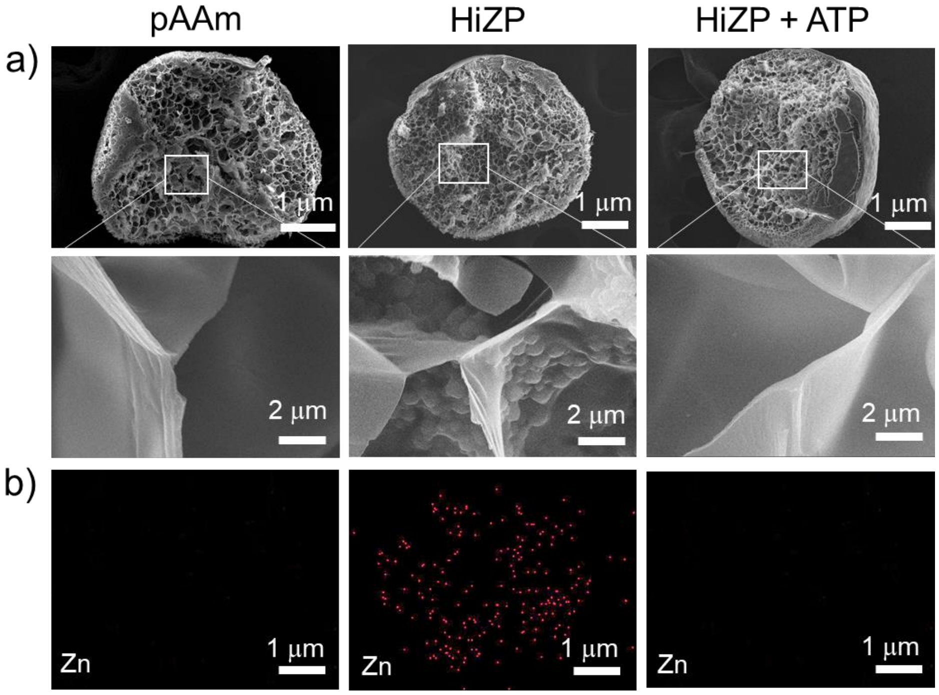

Supplementary Figure 9. Cross-sectional SEM image (a) and EDS mapping of Zn element (b) of pure pAAm hydrogel, HiZP and treated HiZP with ATP. The images are representatives of three independent experiments with similar results.

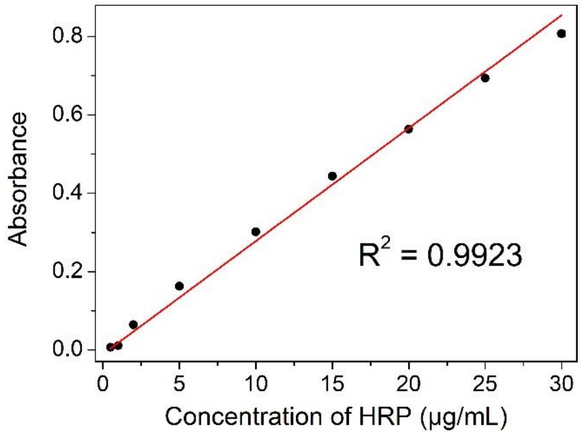

Supplementary Figure 10. The calibration curve of the absorbance of BCA reagent against HRP concentration.

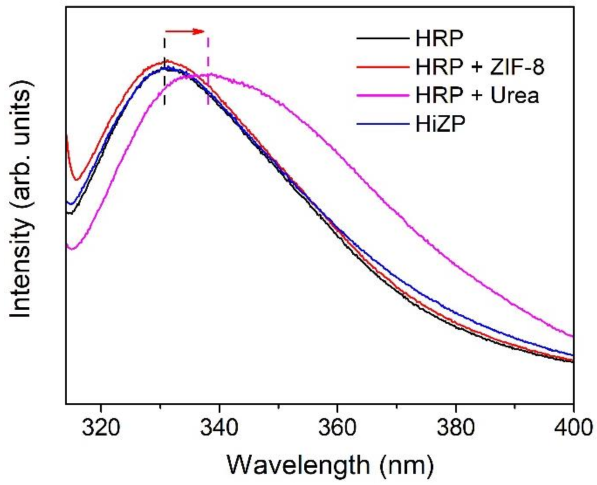

Supplementary Figure 11. Fluorescent emission spectra of HRP, HiZP, treated HRP with 8 M urea, and the mixture of HRP and ZIF-8.

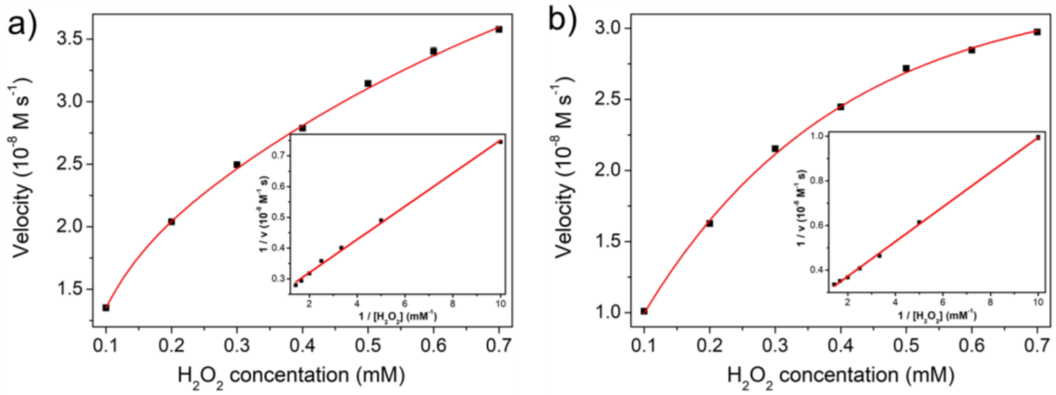

Supplementary Figure 12. Steady-state kinetics of free HRP (a) and HiZP (b). The insets are their corresponding double-reciprocal plots. Data are presented as mean values ± SD (n = 3 independent samples).

The kinetic behaviors of free HRP (a) and HiZP were studied by monitoring the absorbance of $ABTS^{\bullet +}$ in 3-min intervals while varying the concentrations of H_2O_2 and ABTS. The Michaelis-Menten constant was calculated using Lineweaver-Burk plots of the double reciprocal of the Michaelis-Menten equation, $1/v = K_m/V_{max} (1/[S] + 1/K_m)$, where v is the initial velocity, V_{max} represents the maximal reaction velocity, [S] corresponds to the substrate concentration, and K_m is the Michaelis constant. Each sample was measured for three times. All the error bars represent the standard deviations from three repeated experiments.

Supplementary Table 1. Kinetic date of free HRP and HiZP

Catalyst	Substrate	K_m / mM	V_{max} / 10^{-8} M s^{-1}	K_{cat} / s^{-1}
free HRP	H_2O_2	0.253	4.628	7.459
HiZP	H_2O_2	0.307	4.598	7.397

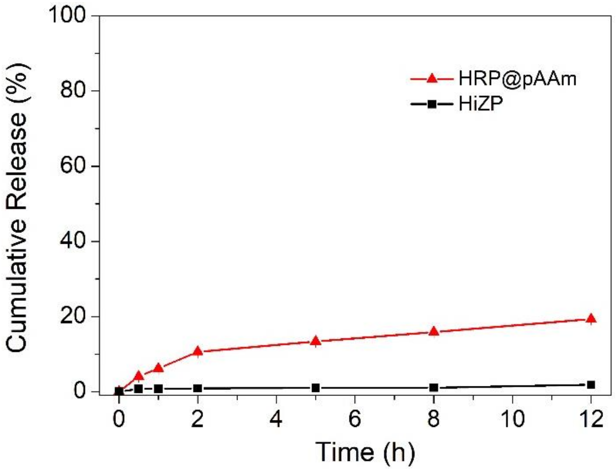

Supplementary Figure 13. Release profiles of HRP from HRP@pAAm and HiZP. Data are presented as mean values ± SD (n = 3 independent experiments).

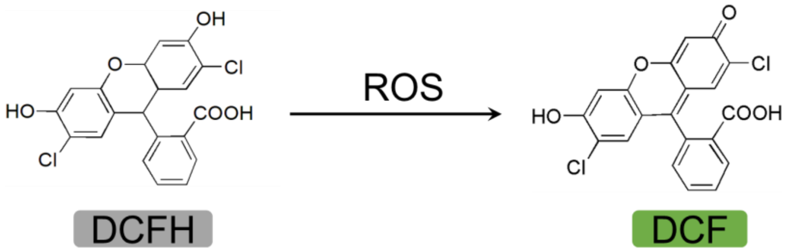

Supplementary Figure 14. Schematic illustration of DCFH oxidation reaction with ROS to produce DCF with strong fluorescence.

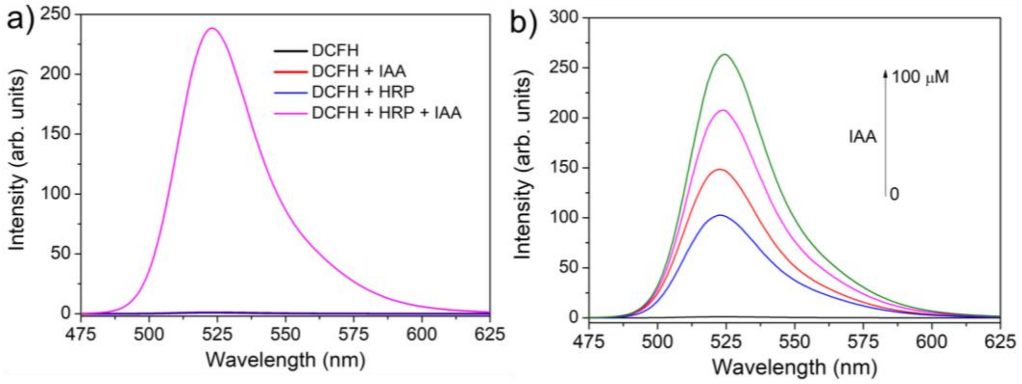

Supplementary Figure 15. (a) Emission spectra of DCFH alone and in the presence of IAA, HRP and the mixture of IAA and HRP. (b) Emission spectra of DCFH in the presence of HRP with different concentrations of IAA.

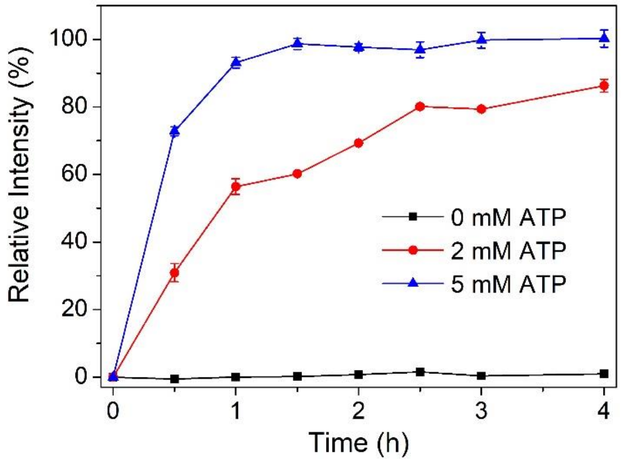

Supplementary Figure 16. Time-dependent fluorescence intensity of DCF at 520 nm in the presence of mixtures of HiZP with different concentrations of ATP. Data are presented as mean values ± SD (n = 3 independent samples).

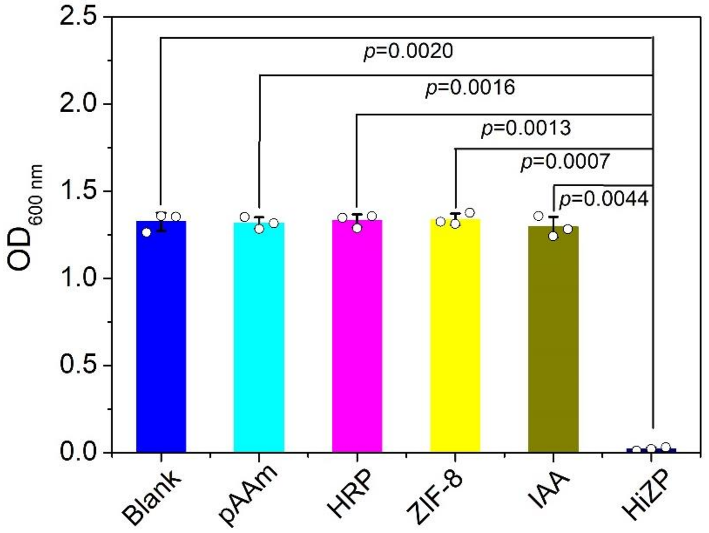

Supplementary Figure 17. The values of the optical density at 600 nm (OD_{600}) of *S. aureus* alone and after incubation with pAAm, HRP, IAA or ZIF-8, and HiZP. Data are presented as mean values ± SD (n = 3 independent experiments). Statistical significance was analyzed via one-way ANOVA with Tukey's multiple comparisons test.

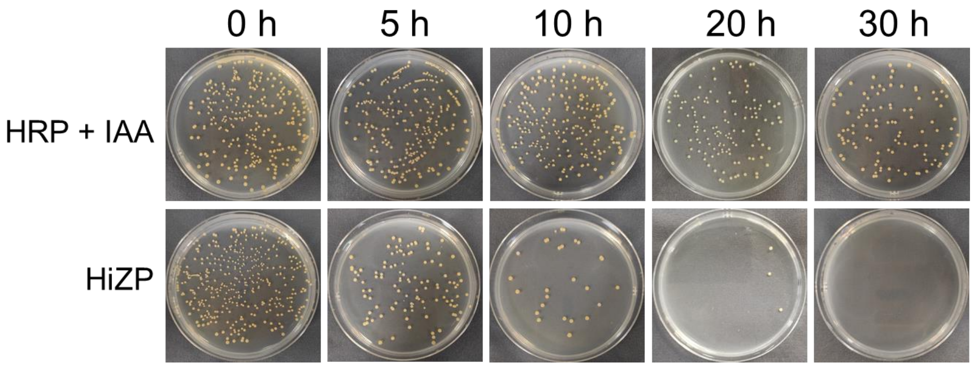

Supplementary Figure 18. Images of *S. aureus* colonies formed on Luria-Bertani agar plates treated with free HRP/IAA system and HiZP.

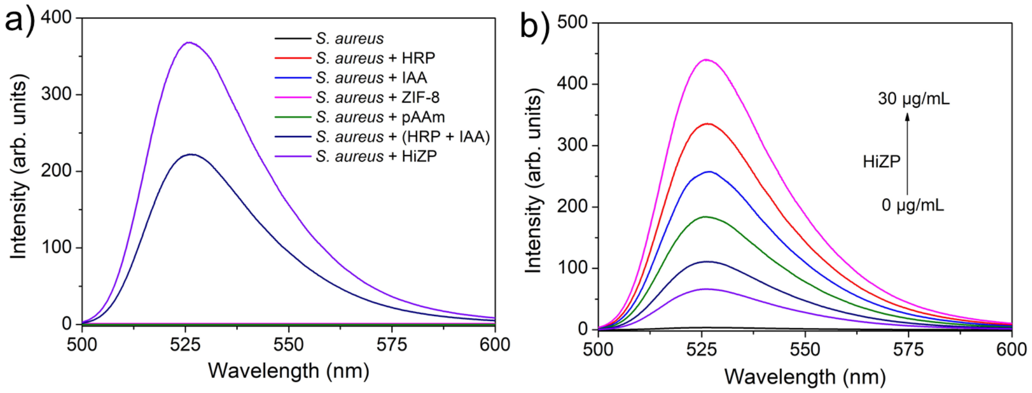

Supplementary Figure 19. Fluorescence spectra of DiBAC4(3) measured from the stained *S. aureus* after different treatments (a) and the stained *S. aureus* after treating with different concentration of HiZP (b).

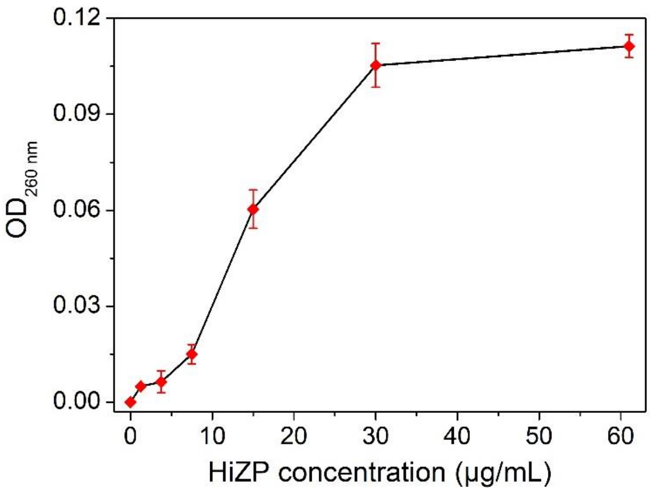

Supplementary Figure 20. The values of optical density at 260 nm (OD_{260}) for evaluation of the leakage of intracellular components from *S. aureus* after incubation with different concentration of HiZP. Data are presented as mean values ± SD (n = 3 independent experiments).

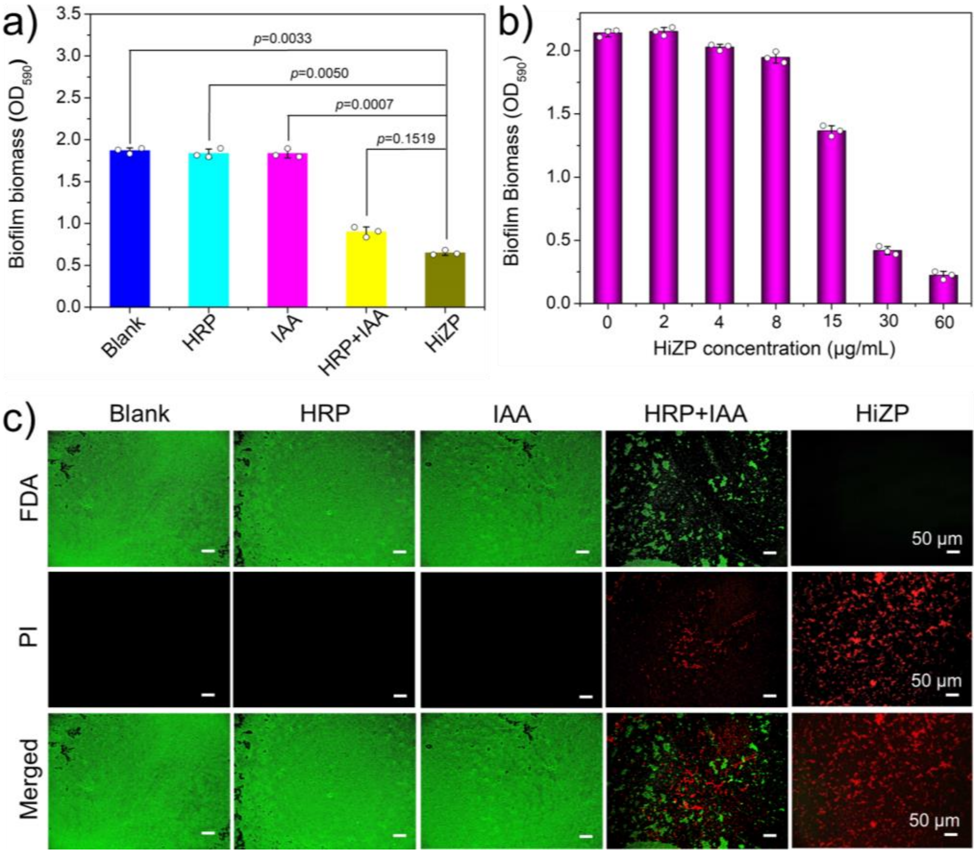

Supplementary Figure 21. OD_{590} values for evaluating the inhibition efficacy of *S. aureus* biofilm after different treatments (a) and treating with different concentration of HiZP (b). Data are presented as mean values ± SD (n = 3 independent experiments). Statistical significance was analyzed via one-way ANOVA with Tukey's multiple comparisons test. (c) Live/dead fluorescent stain images of residual biofilm after different treatments. The images are representatives of three independent experiments with similar results.

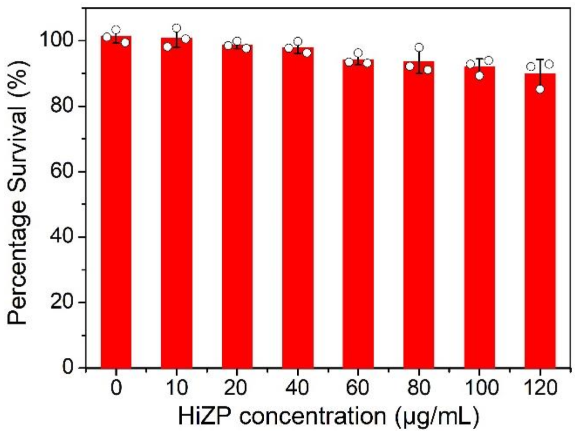

Supplementary Figure 22. Viability of the NIH/3T3 cells treated with different concentrations of HiZP. Data are presented as mean values ± SD (n = 3 independent experiments).

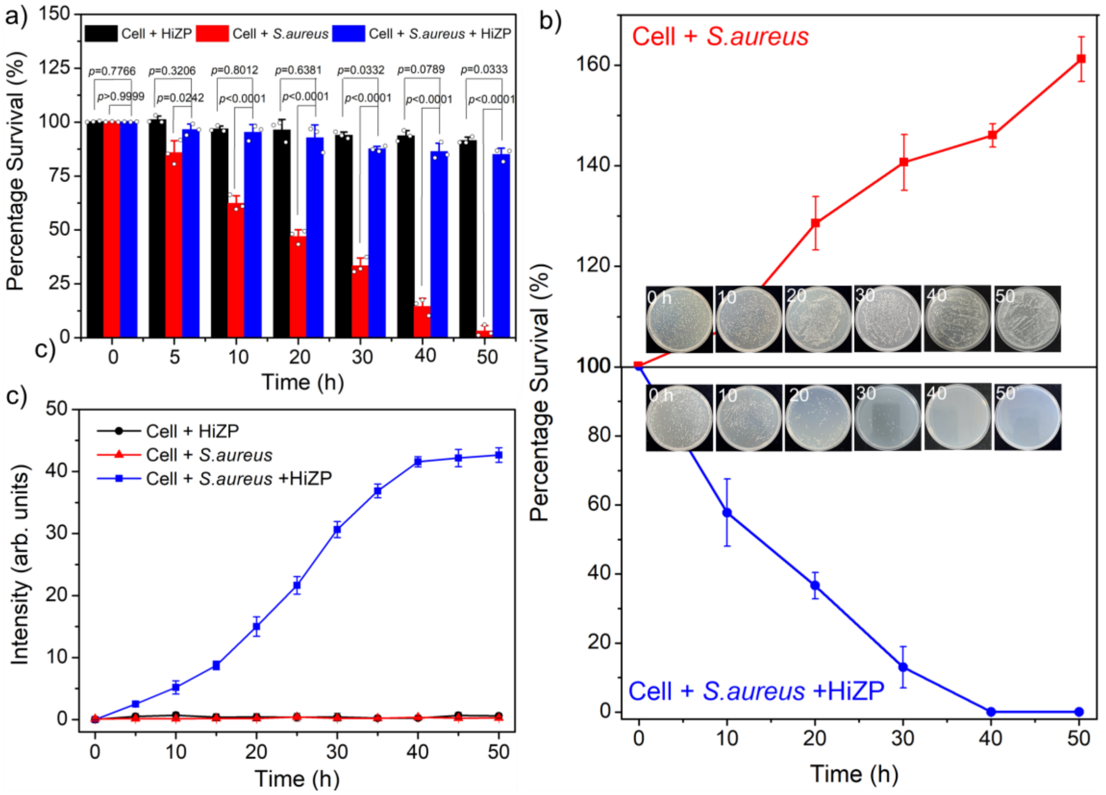

Supplementary Figure 23. (a) Viability of NIH/3T3 cells in the co-culture systems of NIH/3T3 cells with HiZP, *S. aureus*, and *S. aureus* and HiZP. (b) Viability of *S. aureus* in the co-culture systems of NIH/3T3 cells with *S. aureus* (upper) and *S. aureus* and HiZP (lower). (c) Time-dependent DCF fluorescence in the co-culture systems of NIH/3T3 cells with HiZP, *S. aureus*, and *S. aureus* and HiZP. Data are presented as mean values ± SD (n = 3 independent experiments). Statistical significance was analyzed via one-way ANOVA with Tukey's multiple comparisons test.

The co-culture system was constructed by mixing NIH/3T3 cells with *S. aureus* in a 96-well plate, which was incubated in a 5% CO_2 atmosphere. The initial densities of NIH/3T3 cell and *S. aureus* in each well are 6×10^4 cells and 1×10^7 CFU/mL, respectively. The MTT assay results (Supplementary Fig. 23a) show that HiZP has a negligible cytotoxicity against NIH/3T3 cells, demonstrating its excellent biocompatibility. However, the presence of *S. aureus* can seriously affect the survival of NIH/3T3 cells, and almost no surviving cells (< 3%) were observed after 50 h of incubation. Meanwhile, it was found that with the decease of the viability of NIH/3T3 cells, the number of surviving *S. aureus* was remarkably increased in the co-culture system (upper figure of Supplementary Fig. 23b). This is accord with the bacterial contamination effects for cell culture. However, upon the addition of HiZP into the co-culture system, a high viability (> 85%) can be observed in the NIH/3T3 cells, while *S. aureus* shows a decreased surviving rate and was completely killed after 40 h of incubation (lower figure of Supplementary Fig. 23b). This indicates that the addition of HiZP can cause *S. aureus* inactivation to enhance the survival of NIH/3T3 cells, which is consistent with the time-dependent increase of ROS generation in the co-culture system of NIH/3T3 cells with *S. aureus* and HiZP, as reflected by the enhancement of DCF fluorescence in Supplementary Fig. 23c. By contrast, no DCF fluorescence was recorded from the co-culture systems of NIH/3T3 cells with individual HiZP and *S. aureus*, revealing that no ROS was generated in these two systems. Apparently, these results demonstrated the feasibility of using HiZP as a prodrug system for on-demand bacterial inactivation and infection treatment with minimal side effects.

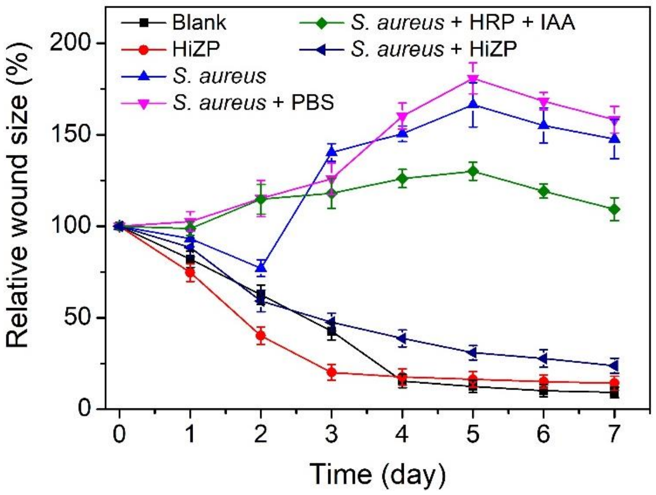

Supplementary Figure 24. The changes of wound size of the mice with different treatments. Data are presented as mean values ± SD (n = 3 independent experiments).

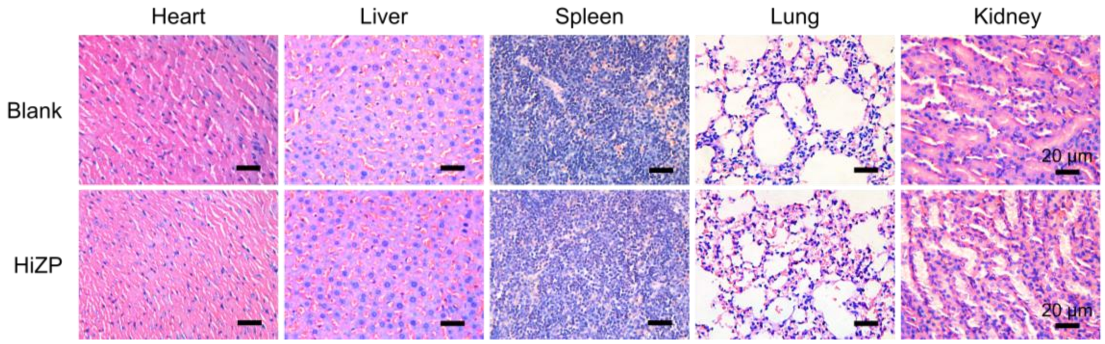

Supplementary Figure 25. Photomicrographs of H&E staining obtained from the major organs (heart, liver, spleen, lung, and kidney) of untreated mice and treated mice with HiZP after 7 days. The images are representatives of three independent experiments with similar results. Scale bars are 20 μm.

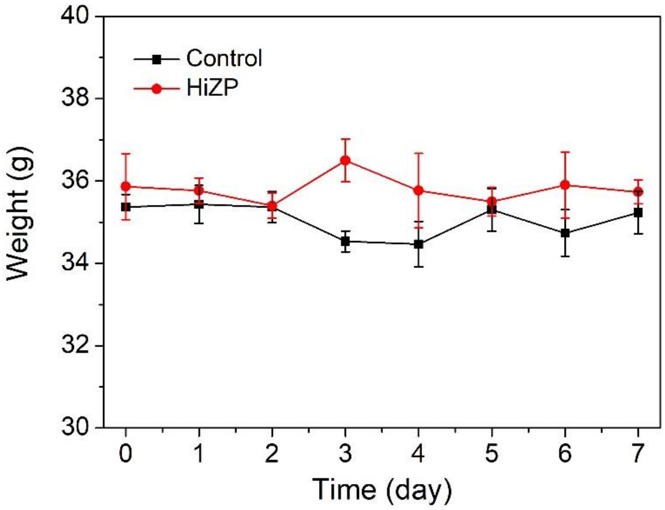

Supplementary Figure 26. Body weights of mice treated with PBS (control) and HiZP. Data are presented as mean values ± SD (n = 3 independent experiments).

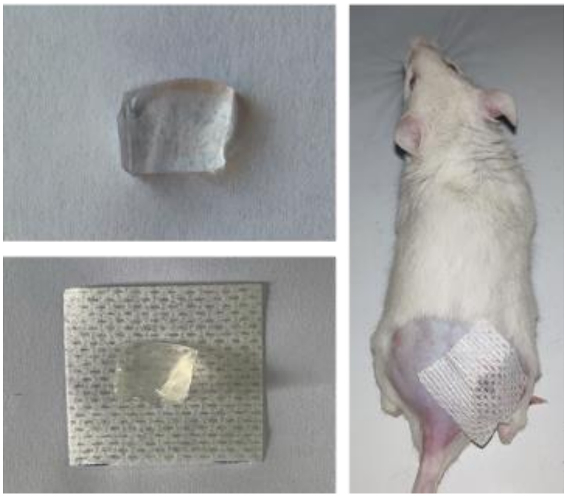

Supplementary Figure 27. Preparation of alginate hydrogel patch for wound healing.
