# Supplementary material for: Adenosine triphosphate-activated prodrug system for on-demand bacterial inactivation and wound disinfection
Source: Nat Commun. 2022 Aug 11;13:4712. doi: 10.1038/s41467-022-32453-3 (PMC9372092; doi:10.1038/s41467-022-32453-3)
Supplement: Supplementary file 3 — Reporting Summary [file 41467_2022_32453_MOESM3_ESM.pdf]

nature portfolio

Corresponding author(s): Hongliang Tan

Last updated by author(s): Jul 10, 2022

Reporting Summary

Nature Portfolio wishes to improve the reproducibility of the work that we publish. This form provides structure for consistency and transparency in reporting. For further information on Nature Portfolio policies, see our Editorial Policies and the Editorial Policy Checklist.

Statistics

For all statistical analyses, confirm that the following items are present in the figure legend, table legend, main text, or Methods section.

n/a	Confirmed	
☐	☒	The exact sample size (n) for each experimental group/condition, given as a discrete number and unit of measurement
☐	☒	A statement on whether measurements were taken from distinct samples or whether the same sample was measured repeatedly
☐	☒	The statistical test(s) used AND whether they are one- or two-sided<br>*Only common tests should be described solely by name; describe more complex techniques in the Methods section.*
☒	☐	A description of all covariates tested
☒	☐	A description of any assumptions or corrections, such as tests of normality and adjustment for multiple comparisons
☐	☒	A full description of the statistical parameters including central tendency (e.g. means) or other basic estimates (e.g. regression coefficient) AND variation (e.g. standard deviation) or associated estimates of uncertainty (e.g. confidence intervals)
☐	☒	For null hypothesis testing, the test statistic (e.g. F, t, r) with confidence intervals, effect sizes, degrees of freedom and P value noted<br>*Give P values as exact values whenever suitable.*
☒	☐	For Bayesian analysis, information on the choice of priors and Markov chain Monte Carlo settings
☒	☐	For hierarchical and complex designs, identification of the appropriate level for tests and full reporting of outcomes
☒	☐	Estimates of effect sizes (e.g. Cohen's d, Pearson's r), indicating how they were calculated

Our web collection on statistics for biologists contains articles on many of the points above.

Software and code

Policy information about availability of computer code

Data collection	Fluorescence spectra were collected using a fluorescence spectrophotometer (Hitachi, F-7000, Japan).<br>UV-vis absorbance spectra were collected using a UV-Vis spectrophotometer (Hitachi, U-3900H, Japan).<br>Optical images of hydrogel microsphere, fluorescence staining of bacteria and biofilms, H&E staining, and Masson staining were acquired using an inverted fluorescence microscope (Olympus, IX53, Japan).<br>Confocal laser scanning microscope images were collected using a confocal microscope (Leica, TCS SP8, Germany).<br>SEM images were collected using a scanning electron microscope (Hitachi, S-3400, Japan).<br>Optical density was measured using a microplate reader (Molecular Devices, SpectraMax M2, USA).
Data analysis	Origin 8.0 and Graphpad Prism 8 were used for all statistical analysis. Image J version 1.53c was used to contrast and overlay images.

For manuscripts utilizing custom algorithms or software that are central to the research but not yet described in published literature, software must be made available to editors and reviewers. We strongly encourage code deposition in a community repository (e.g. GitHub). See the Nature Portfolio guidelines for submitting code & software for further information.

Data

Policy information about availability of data

All manuscripts must include a data availability statement. This statement should provide the following information, where applicable:

- Accession codes, unique identifiers, or web links for publicly available datasets
- A description of any restrictions on data availability
- For clinical datasets or third party data, please ensure that the statement adheres to our policy

The data generated in this study are provided in the Supplementary Information and the Source Data file provided with this paper. Data is also available from the

corresponding author upon request.

Field-specific reporting

Please select the one below that is the best fit for your research. If you are not sure, read the appropriate sections before making your selection.

- [x] Life sciences
- [ ] Behavioural & social sciences
- [ ] Ecological, evolutionary & environmental sciences

For a reference copy of the document with all sections, see nature.com/documents/nr-reporting-summary-flat.pdf

Life sciences study design

All studies must disclose on these points even when the disclosure is negative.

Sample size	No statistical methods were used to predetermine sample size, but the sample sizes employed in this study are consistent with previously published works. For example, all experiments were repeated three times independently and the in vivo experiments with 3 mice each group were used.
Data exclusions	No data were excluded.
Replication	All experiments were repeated three times independently and experimental findings were reproducible.
Randomization	All samples were randomly allocated into experimental groups.
Blinding	The investigators normally were not blinded to group allocation during data collection and/or analysis, but unbiased experimental procedure and data collect and/or analysis were carried out as far as possible.

Reporting for specific materials, systems and methods

We require information from authors about some types of materials, experimental systems and methods used in many studies. Here, indicate whether each material, system or method listed is relevant to your study. If you are not sure if a list item applies to your research, read the appropriate section before selecting a response.

Materials & experimental systems

n/a	Involved in the study	
x		Antibodies
	x	Eukaryotic cell lines
x		Palaeontology and archaeology
	x	Animals and other organisms
x		Human research participants
x		Clinical data
x		Dual use research of concern

Methods

n/a	Involved in the study	
x		ChIP-seq
x		Flow cytometry
x		MRI-based neuroimaging

Eukaryotic cell lines

Policy information about cell lines

Cell line source(s)	NIH/3T3 cell line were obtained from China Center for Type Culture Collection.
Authentication	Cells were not authenticated.
Mycoplasma contamination	No mycoplasma contamination was found.
Commonly misidentified lines (See ICLAC register)	No commonly misidentified cell lines were used.

Animals and other organisms

Policy information about studies involving animals; ARRIVE guidelines recommended for reporting animal research

Laboratory animals	Kunming mice (male, 30-40 g) with 8 weeks old were used for wound healing and organs (heart, liver, spleen, lungs, and kidneys) collection. The mice were bought from Laboratory Animal Science and Technology Center at Jiangxi University of Chinese Medicine and were raised in the pathogen-free room with a temperature of 25 ± 3 °C, a relative humidity of 60 - 70%, and a 12 h light/dark cycle.
Wild animals	This study did not involve wild animals.

Field-collected samples	This study did not involve samples collected from the field.
Ethics oversight	All animal experimental procedures were in accordance with the guidelines of the National Institutes of Health for the Care and Use of Laboratory Animals and were approved by the Ethics Committee of College of Life Science at Jiangxi Normal University (No. 20211023001).

Note that full information on the approval of the study protocol must also be provided in the manuscript.
